# Influence of Corpus Callosum Damage on Cognition and Physical Disability in Multiple Sclerosis: A Multimodal Study

**DOI:** 10.1371/journal.pone.0037167

**Published:** 2012-05-14

**Authors:** Sara Llufriu, Yolanda Blanco, Eloy Martinez-Heras, Jordi Casanova-Molla, Iñigo Gabilondo, Maria Sepulveda, Carles Falcon, Joan Berenguer, Nuria Bargallo, Pablo Villoslada, Francesc Graus, Josep Valls-Sole, Albert Saiz

**Affiliations:** 1 Center for Neuroimmunology, Service of Neurology, Hospital Clinic and Institut d'Investigacions Biomèdiques August Pi i Sunyer (IDIBAPS), Barcelona, Spain; 2 Service of Neurology, Hospital Clinic, Barcelona, Spain; 3 Medical Imaging Platform, Institut d'Investigacions Biomèdiques August Pi i Sunyer (IDIBAPS), Barcelona, Spain; 4 Centros de Investigación Biomédica en Red (CIBER-BBN), Barcelona, Spain; 5 Service of Radiology and Imaging Diagnostic Center, Hospital Clinic and Institut d'Investigacions Biomèdiques August Pi i Sunyer (IDIBAPS), Barcelona, Spain; Charité University Medicine Berlin, Germany

## Abstract

**Background:**

Corpus callosum (CC) is a common target for multiple sclerosis (MS) pathology. We investigated the influence of CC damage on physical disability and cognitive dysfunction using a multimodal approach.

**Methods:**

Twenty-one relapsing-remitting MS patients and 13 healthy controls underwent structural MRI and diffusion tensor of the CC (fractional anisotropy; mean diffusivity, MD; radial diffusivity, RD; axial diffusivity). Interhemisferic transfer of motor inhibition was assessed by recording the ipsilateral silent period (iSP) to transcranial magnetic stimulation. We evaluated cognitive function using the Brief Repeatable Battery and physical disability using the Expanded Disability Status Scale (EDSS) and the MS Functional Composite (MSFC) z-score.

**Results:**

The iSP latency correlated with physical disability scores (r ranged from 0.596 to 0.657, *P* values from 0.004 to 0.001), and with results of visual memory (r = −0.645, *P* = 0.002), processing speed (r = −0.51, *P* = 0.018) and executive cognitive domain tests (r = −0.452, *P* = 0.039). The area of the rostrum correlated with the EDSS (r = −0.442, *P* = 0.045). MD and RD correlated with cognitive performance, mainly with results of visual and verbal memory tests (r ranged from −0.446 to −0.546, *P* values from 0.048 to 0.011). The iSP latency correlated with CC area (r = −0.345, *P* = 0.049), volume (r = −0.401, *P* = 0.002), MD (r = 0.404, *P* = 0.002) and RD (r = 0.415, *P* = 0.016).

**Conclusions:**

We found evidence for structural and microstructural CC abnormalities associated with impairment of motor callosal inhibitory conduction in MS. CC damage may contribute to cognitive dysfunction and in less extent to physical disability likely through a disconnection mechanism.

## Introduction

The corpus callosum (CC) is the major cerebral commissure. It connects homologous regions of both sides of the brain providing interhemispheric communication between cortical and subcortical neurons. It plays an important role in the organization of complex commands involving bilateral tasks with precise timing of information transfer between sides [1]. The CC is a common target in multiple sclerosis (MS) frequently showing focal demyelinating lesions and atrophy since early stages of the disease [2]. Hence it is of interest to study whether CC damage influences cognitive impairment and physical disability in MS, two of the most devastating consequences of the disease.

The association between disability measured by the Expanded Disability Status Scale (EDSS) [3] and CC atrophy remains inconsistent, with some studies showing a positive correlation [2], [4], but others reported no significant relationship [5], [6]. In contrast to structural magnetic resonance imaging (MRI) techniques, the evaluation of CC by means of diffusion tensor imaging (DTI) seems to correlate better with clinical measures of disability [6], [7].

Only few studies have addressed the relationship between CC damage and cognitive impairment in MS. A significant correlation has been reported between CC atrophy and the severity of impairment in the performance of tasks requiring interhemispheric transfer of information [8] and also between a number of diffusion metrics from tractography maps or DTI voxel-based approaches and the results of a single cognitive test [9], [10] or a battery of neuropsychological tests [11], [12]. In overall, the revised literature suggests that cognitive impairment in MS could be the consequence of disconnection between cortical and subcortical circuits [13].

**Figure 1:**
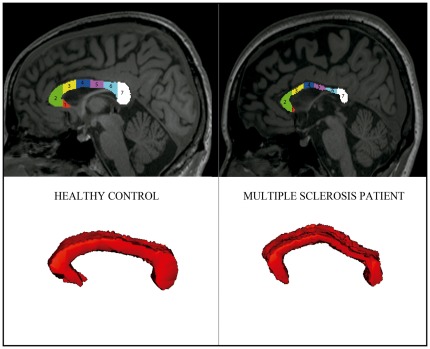
Structural magnetic resonance imaging of the corpus callosum. Area of corpus callosum in midsagittal slice with subdivision in 7 segments, corresponding consecutively to the rostrum, genu, rostral body, anterior midbody, posterior midbody, isthmus, and the splenium (top images) and volume obtained from 11 consecutive central sagittal slices (bottom images). Left images correspond to a healthy control and right images to a multiple sclerosis patient.

The interhemispheric connectivity through the CC can be evaluated by means of transcranial magnetic stimulation techniques (TMS), i.e. interhemispheric inhibition (IHI) and ipsilateral silent period (iSP) [14], [15], [16]. A recent study found that even with no evidence of macroscopic CC lesions, patients with early relapsing-remitting MS showed abnormally reduced IHI and abnormally reduced fractional anisotropy (FA) from DTI within the motor part of the CC, although the correlation between both measures could not be demonstrated in those patients [17]. However, in healthy controls there was a direct linear correlation between IHI and DTI measures [18] suggesting a close link between microstructure and function.

The iSP reflects the inhibition induced on ipsilateral voluntary activity by focal unilateral TMS [16]. It is thought to be mediated through the CC although other more caudal pathways may also exist [19]. Correlation studies looking at iSP, physical disability and structural CC MRI changes have shown conflicting results [20], [21], [22]. However, the relationship between iSP and microstructural CC damage or cognition has not been investigated so far. DTI allows the study of microscopic Brownian motion of water molecules hindered by cellular structures, such as cell membranes and axonal cytoskeleton. This property makes the technique sensitive to microstructure integrity disruption, frequent in MS lesions and in normal-appearing white matter (NAWM) [11], [23]. White matter damage is reflected by a reduction in FA and increase in overall diffusion (mean diffusivity, MD), which are non-specific metrics due to the various underlying pathophysiological mechanisms [24]. Other metrics derived from FA are axial diffusivity (AD) and radial diffusivity (RD), which seem to be related to axonal damage and demyelination respectively in animal models [25].

With this background we hypothesized that the cognitive impairment and physical disability in MS could be associated with disconnection between brain areas due to CC damage. To test this hypothesis we used a multimodal approach to investigate CC status by analyzing the structural/microstructural damage of the CC, and the function of motor inhibitory connections through the CC by the iSP in a series of relapsing-remitting MS patients. Then, we analyzed the relationship between the extent of CC damage and cognitive impairment, using a battery of neuropsychological tests, and scores on standard disability scales including the EDSS and the Multiple Sclerosis Functional Composite (MSFC) [26].

## Methods

### Study subjects

Twenty one consecutive patients with relapsing-remitting MS [27] were prospectively selected from the outpatient MS Clinic of the Hospital Clinic of Barcelona. All patients had to be ambulatory with low to moderate disability (EDSS 0 to 6.0), under stable immunomodulatory treatment and relapse and steroid-free for at least one month prior to the study inclusion. Thirteen age and sex matched healthy individuals were included as controls. The Hospital Clinic Research Ethics Committee approved the study and all participants gave written informed consent.

### Clinical and cognitive evaluation

The disease history was recorded and a complete neurological examination including the EDSS and MSFC z-score punctuation to assess physical disability was obtained. The z-score of the MSFC was calculated by comparison with a standard MS population according to the protocol of the National MS Society [26]. The Brief Repeatable Battery–Neuropsychology test (BRB-N) was used to assess the cognitive status. Neuropsychological test scores were expressed as z-scores for all tests and cognitive domains, derived from normative data obtained from a published Spanish healthy control cohort study and stratified by age and education [28]. Tests included in the BRB-N were: the Selective Reminding Tests (SRT) to assess verbal memory; the 10/36 Spatial Recall Tests (10/36) to assess visual memory; the Symbol Digit Modalities Test (SDMT) to assess attention, visual precision search, processing speed and executive functions; the Paced Auditory Serial Addition Task 3 seconds (PASAT 3) to assess the maintenance of attention, processing speed and working memory; and the World List Generation (WLG) to assess associative verbal fluency. The z-scores from the verbal memory domain, visual memory domain, attentional/executive domain, fluency domain, and the global BRB-N were measured as previously reported [28]. Physical and cognitive scores were not measured in control subjects.

### Neurophysiological evaluation

TMS was used to assess motor threshold (passive and active), central motor conduction time and ipsilteral silent period in hand muscles. A Mystro 5Plus EMG was used for recording the responses and triggering the magnetic stimulator. The stimulator was a Novametrix 200 (MagStim, London), equipped with an 8-shaped coil for cortical stimulation and a round coil for cervical foraminal stimulation. Surface recording electrodes were attached over the first dorsal interosseous (FI) muscles of both sides. Resting motor threshold was measured as the minimum stimulus intensity needed to elicit motor evoked potentials (MEPs) of at least 50 µV in 50% of a series of stimuli [29]. The stimulus intensity was changed stepwise in 1% increment or decrement until the threshold was reached. We applied magnetic stimulation to the scalp and to the cervical region. During cortical stimulation, subjects were requested to maintain a slight preactivation of the FI. The latency difference between the MEPs to cortical and cervical stimulation was calculated as the central motor conduction time (CMCT). Then, subjects were asked to keep a steady contraction of about 20% of their maximum voluntary contraction by pressing the index finger against the thumb in both hands. Stimuli of suprathreshold intensity were applied six times in each side. The mean duration and onset latency of the iSP from both hands were manually measured by a technician unaware of clinical and MRI data.

**Table 1:** Results from the cognitive tests and domains included in the Brief Repeatable Battery in patients with multiple sclerosis.

Tests	SRTS	SRTR	SRTD	10/36	10/36D	SDMT	PASAT	WLG
	−0.68 (1.27)	−0.80 (1.21)	−0.03 (1.06)	−0.14 (0.75)	−0.03 (1.15)	0.21 (1.24)	−0.53 (1.16)	−1.13 (0.77)

Cognitive domains	Verbal memory	Visual memory	Attentional/executive	Fluency
	−0.64 (1.14)	−0.09 (0.81)	−0.15 (1.12)	−1.13 (0.77)
**Global BRB**	−0.50 (0.77)			

Values are mean ± SD z-scores. Abbreviations: SRTS = selective reminding test long term storage; SRTR = selective reminding test long term retrieval; SRTD = selective reminding test delayed recall; 10/36 = spatial recall test; SDMT = symbol digit modalities test; PASAT-3 = paced auditory serial addition task 3 seconds; WLG = world list generation; BRB = Brief Repeatable Battery.

### Image acquisition and data postprocessing

All scans were performed on a 3T Siemens Trio MRI scanner (Erlangen, Germany), using a 32 channel head coil for radio frequency transmission and signal reception. The MRI protocol included the following sequences: a) 3D structural T1-weighted MPRAGE (Magnetization Prepared Rapid Acquisition Gradient Echo) sequence: Repetition Time (TR): 2050 ms, Echo Time (TE): 2.4 ms, Inversion recovery time (TI): 1050 ms, Flip angle: 9°, FOV: 220 mm; b) DTI sequence: TR/TE: 7600/89 ms, acquisition matrix 122×122, FOV: 250 mm, 60 contiguous axial slices, diffusion gradients in 30 different directions, b value = 1000 s·mm^−2^.

CC area was calculated from the 3D structural T1-weighted midsagittal slice by means of semiautomatic segmentation tool of Analyze software 9.0 (http://www.analyzedirect.com; Biomedical Imaging Resource, Mayo Clinic). The midsagittal CC section was then divided in 7 segments with a semiautomated subregional division [30] (Figure 1). A CC mask was drawn in 11 consecutive slices around the midsagittal plane where it could be clearly differentiated from the cingulated gyrus. Moreover, all the CC lesions were manually traced on the MPRAGE sequence to get a lesion load from the CC. The volumetric and DTI analysis of the CC included the visible lesions. The volume of the CC mask was calculated by adding the area of the 11 consecutive slices (area per slice thickness) (Figure 1). The CC area and the volume were then multiplied by a brain scaling factor calculated by SIENAX (FMRIB, Oxford, UK) to normalize for the cranial size.

An experienced neurologist created T1-lesion masks using semi-automated thresholding and manual editing methods directly from the high-resolution T1-MPRAGE images. Subsequent brain segmentation and normalization were performed using SIENAX, which was effectively fully automated once the T1 lesion mask had been used to avoid pixel misclassifications. The following parameters were derived from the segmentation: the normalized brain parenchymal volume (nBPV), normal-appearing white matter volume (nWMV), grey matter volume (nGMV) and whole brain T1-MPRAGE lesion volume (nLV).

FSL (FMRIB, Oxford, UK) tools were used to register DTI to structural images. A linear (FLIRT, solid rigid transformation) and nonlinear (FNIRT) registrations of FA maps to skull removed structural image were performed excluding the CC lesions when estimating the warps. The double registration was done aiming to consider changes in displacement and shape with high accuracy to structural image. The resulting transformation files were applied to index images derived from DTI to coregister them to CC mask. The borders of the CC mask were eroded to avoid that partial volume voxels diminishing mean values of the diffusivity indexes. The mean values of the following parameters were obtained in CC: FA, MD, RD (the average of the second and third eigenvalues) and AD (the first eigenvalue) from the CC mask.

### Statistical analysis

The statistical analysis was performed using SPSS for Windows (version 17.0). Normality was assessed by the Kolmogorov-Smirnov test; all the variables except for the CC lesion volume had a normal distribution. All values are expressed as mean (SD). The independent-samples Student's *t* test was used to compare demographic, neurophysiologic and MRI metrics between MS and controls. Univariate correlations between clinical, neurophysiological, cognitive and MRI data were examined in patients with the Pearson correlation coefficients. A linear regression model was used to evaluate the effect of disease duration and disability on iSP measures. *P*-values<0.05 were considered to indicate statistical significance.

## Results

### Clinical and cognitive data

The 21 MS patients had a mean (SD) age of 37.2 (6.9) years, 12 (57%) were female with mean disease duration of 9.5 (5.38) years. Median EDSS score was 2.0 (range 0 to 6.0) and mean MSFC z-score 0.23 (0.59). The 13 healthy controls had a mean age of 35.2 (7.4), similar to the age of the patients (*P* = 0.45), and 8 (61.5%) were females. The results of the cognitive tests in the cohort of MS patients are shown in Table 1.

### Neurophysiological study

A summary of the data extracted from the TMS study is shown in Table 2. The mean iSP onset latency was significantly longer in MS patients than in controls (*P* = 0.001). There was a statistically significant correlation between iSP latency and the EDSS score (r = 0.657, *P* = 0.001), the MSFC z-score (r = −0.596, *P* = 0.004), and cognitive data, i.e., the 10/36 (r = −0.645, *P* = 0.002), the SDMT (r = −0.51, *P* = 0.018) and the executive cognitive domain (r = −0.452, *P* = 0.039) z-scores. Moreover, the iSP latency correlated with disease duration, but this effect disappeared (B = 0.170, *P* = 0.426) when the EDSS sore was added to the model (B = 0.564, *P* = 0.015).

**Table 2:** Results from the ipsilateral silent period and central motor conduction time.

	MS patients	Controls	*P*
iSP latency	41.25 (4.95)	35.47 (3.62)	0.001
iSP duration	20.06 (4.71)	18.51 (2.67)	0.25
CMCT latency	8.51 (2.60)	6.20 (1.11)	0.007

Values are mean (SD). Results from the electrophysiology study are expressed in milliseconds. Abbreviations: CMCT = central motor conduction time; iSP = ipsilateral silent period; MS = multiple sclerosis.

The mean iSP duration was not different between MS patients and controls. We did not find any significant correlation between the duration of the iSP and the physical disability or cognitive dysfunction. The latency of the MEP to cortical stimulation was significantly longer in patients than in controls subjects, resulting in a longer mean CMCT (*P* = 0.007). There was no significant correlation between CMCT latency and physical disability (EDSS: r = 0.248, *P* = 0.278; MSFC: r = −0.028, *P* = 0.905) or cognitive data (data not shown). However, a significant correlation was found between the CMCT and the iSP latency (r = 0.398, *P* = 0.022). Finally, there were no significant differences in resting motor threshold between patients and control subjects (*P*>0.05).

### Corpus callosum MRI data

Table 3 summarizes the data concerning MRI findings. There were no significant differences in the area of the CC in the midsagittal slice between MS patients and controls, although the area of segment 1 (rostrum of the CC), but not of the other segments, was smaller in patients (*P* = 0.03). The CC volume was smaller in MS patients compared with controls (*P* = 0.007). The CC volume correlated with the nLV (r = −0.686, *P* = 0.001). The area of segment 1 correlated with the EDSS (r = −0.442, *P* = 0.045) and with the 10/36 delayed (r = 0.439, *P* = 0.047) test. The other CC structural measures did not significantly correlate with physical disability or cognitive data (data not shown).

**Table 3:** Results from the corpus callosum and the whole brain structural MRI analysis.

	MS patients	Controls	*P*
Area of CC (midsagittal slice)	6.78 (1.2)	7.11 (0.9)	0.17
Segment 1 area	0.25 (0.1)	0.34 (0.1)	0.03
Segment 2 area	1.55 (0.3)	1.58 (0.3)	0.78
Segment 3 area	0.87(0.2)	0.96 (0.1)	0.20
Segment 4 area	0.84 (0.2)	0.95 (0.09)	0.89
Segment 5 area	0.80 (0.2)	0.78 (0.1)	0.76
Segment 6 area	0.88 (0.2)	0.91 (0.2)	0.68
Segment 7 area	1.60 (0.3)	1.69 (0.3)	0.43
Volume of CC	8.19 (1.65)	9.84 (1.60)	0.007
Volume of CC lesions	0.14 (0.27)	n/a	n/a
nBPV	1,508.8 (72.0)	1,627.3 (81.2)	<0.001
nWMV	725.8 (49.2)	781.7 (32.6)	0.001
nGMV	783 (39.8)	845.6 (57.5)	0.001
nLV	6.99 (8.04)	n/a	n/a

Values are mean (SD). Results from the corpus callosum areas are expressed in cm^2^ and volumes in cm^3^. Abbreviations: CC = corpus callosum; n/a = not applicable; nBPV = normalized brain parenchymal volume; nWMV = normalized normal-appearing white matter volume; nGMV = normalized grey matter volume; nLV = normalized whole brain T1-MPRAGE lesion volume; MS = multiple sclerosis.

Compared with controls, MS patients showed lower FA (P = 0.032) and higher MD (*P* = 0.001), AD (*P* = 0.022) and RD (*P* = 0.003) values. Table 4 shows the results from the DTI analysis.

**Table 4:** Diffusion-tensor MRI-derived metrics of the corpus callosum.

	MS patients	Controls	*p*
Average FA	0.69 (0.04)	0.72 (0.02)	0.03
Average MD	0.97 (0.04)	0.89 (0.03)	0.001
Average AD	1.86 (0.09)	1.78 (0.05)	0.02
Average RD	0.52 (0.03)	0.44 (0.03)	0.003

Values are mean (SD). They refer to metrics of the corpus callosum mask including visible lesions. Average MD, AD and RD are expressed in units of mm^2^/s×10^−3^, FA is a dimensionless index. Abbreviations: AD = axial diffusivity; MD = mean diffusivity; FA = fractional diffusivity; RD = radial diffusivity; MS = multiple sclerosis.

There was no significant correlation between DTI measures and physical disability (EDDS and MSFC z-score). On the other hand, MD values correlated with the 10/36 delayed test (r = −0.546, *P* = 0.011), the verbal memory domain (r = −0.446, *P* = 0.043) and the visual memory domain (r = −0.468, *P* = 0.033), RD values with some cognitive tests, i.e. the SRT long-term storage (r = −0.437, *P* = 0.048), the SRT long-term retrieval (r = −0.484, *P* = 0.026), the 10/36 delayed test (r = −0.502, *P* = 0.02) and the verbal memory domain (r = −0.484, *P* = 0.026).

### Combination of electrophysiological and MRI data

The iSP latency correlated with the area of the CC (r = −0.345, *P* = 0.049), with the area of segments 1 (r = −0.523, *P* = 0.002) and 3 (r = −0.359, *P* = 0.04) and with the volume of the CC (r = −0.401, *P* = 0.021). The iSP onset latency also correlated with whole brain volume metrics, i.e. the nBPV (r = −0.542, *P* = 0.01), the nWMV (r = −0.527, *P* = 0.002) and the nGMV (r = −0439, *P* = 0.011) but the correlation was not significant with the nLV or the lesion load inside of the CC (data not shown). Finally, the iSP latency also correlated with MD (r = 0.404, *P* = 0.02) and RD (r = 0.415, *P* = 0.016) values. The CMCT did not show any correlation with structural or DTI measures (data not shown).

## Discussion

In this study, we investigated the influence of CC damage on the cognitive dysfunction and physical disability in MS patients, using a multimodal approach. First, we assessed the structural and microstructural characteristics of the CC by MRI and the function of motor inhibitory connections through the CC by the iSP. Then, we examined the relationship between the extent of CC damage and cognitive dysfunction using a battery of neuropsychological tests, and physical disability based on standard disability scales. Our study shows that the microstructural integrity of the CC and the motor callosal conduction are impaired in MS patients and these abnormalities correlate with cognitive dysfunction, mainly verbal and visual memory, information processing speed and executive tasks, and in a lesser extent, with physical disability.

It is well known that stimulation of one hemisphere by TMS inhibits the activity generated in the contralateral hemisphere [14], [16]. However, the physiology of this effect is not completely understood. A silent period is produced in the ongoing voluntary activity in both hands to unilateral stimulation. While the contralateral one follows the generation of a MEP and, therefore, it involves post firing of alpha motoneurons, the iSP is never preceded by a MEP and, consequently, it must be considered as the result of suppression of the voluntary corticospinal drive. It is conceivable that the iSP depends on the integrity of transcallosal inhibitory fibers between motor cortices. Increased latency of the iSP in MS patients has been found in some studies [31], [32] and prolonged iSP duration in others [20], [21], [22]. These abnormalities are probably related to demyelination [32]. In the current study the iSP latency was longer in MS patients than in healthy controls, and the same was observed in the 2 patients who did not have evidence of CC lesions (data not shown), revealing motor callosal conduction deficit in MS patients. Although the iSP latency correlated with the duration of the disease, this effect disappeared when the EDSS was added to the statistical model, suggesting that the slowness in the interhemispheric inhibitory command could be a characteristic feature of late disease stages [21] due to the higher physical disability related to those stages.

In agreement with previous studies we found a significant, although moderate, correlation between iSP abnormalities and the CMCT [20]. Although the iSP measures could be in part reflecting the demyelination of the contralateral corticospinal tract, the fact that the iSP but not the CMCT significantly correlated with structural CC damage (atrophy) and with DTI markers of microstructure integrity (MD and RD) would suggest that the CC is a main contributor to this abnormality.

As expected, CC atrophy [6], [8] was found in our patients with non-disabling MS [33]. However, differences with respect to healthy subjects were only significant for the area of the rostrum and the volume of the CC. In line with other reports, the overall callosal volume did not correlate with measures of physical disability [6], although a significant but moderate correlation was found with the area of the rostrum, which is composed by axonal fibers that connect ventro-prefrontal cortices of either hemisphere. In the same way, DTI measures of axonal damage and demyelination, reduced FA and increased MD, AD and RD were also found [17], [32], even though none of them correlated with the physical disability observed in our patients. Our findings are in line with those found in pathological studies showing a substantial loss of both axonal density and volume in the NAWM of the CC with a trend to higher decrease in the rostrum and midbody [34].

Latency of the iSP significantly correlated with scores on standard disability scales including the EDSS and z-MSFC. This is in agreement with previous studies that showed that the functional impairment of interhemispheric transfer analyzed by neuropsychological testing or IHI correlated with the EDSS [8], [22]. Therefore, it is likely that the increased latency of the iSP found in our patients reflects dysfunction of inhibitory fibers connecting the primary motor cortices [35]. In this sense, a previous study found an inverse correlation between iSP duration and the amount of ipsilateral motor cortex activation in functional MRI suggesting that the increased activation represented a consequence of loss of transcallosal inhibitory fibers [32].This would lead to reduced inhibitory input and, consequently, to impairment of the motor performance [32].

The latency of the iSP significantly correlated with the performance of immediate visual spatial memory, working memory, speed of information processing, attention and executive tasks, while some values from the CC microstructure, MD and RD, correlated with immediate and delayed verbal and visual spatial memory, that is with the profile of neuropsychological dysfunction most frequently reported in MS [13], [36], [37]. To the best of our knowledge, the relationship between iSP and cognitive performance has not been previously evaluated in MS patients. Interestingly, though, abnormalities in the iSP have been associated with decrease of verbal fluencies, attention functions and MRI atrophy of the CC in patients with corticobasal degeneration [38].

Previous studies in MS have shown significant correlations between cognitive status and CC microstructure. A significant increase in CC MD values was observed in relapsing-remitting MS patients with cognitive impairment [9], even in the benign MS form [12]. Moreover, patterns of tract FA reduction for cognitive tests, including localizations such as the body and splenium of the CC, only partially overlapped with the visible T2 lesions, supporting that NAWM abnormality contributes to cognitive dysfunction [13].

Our study would favor the hypothesis that, in MS, cognitive impairment, and likely also physical disability, are, at least in part, influenced by the CC status and might result from a multiple disconnection syndrome [13], [39]. Post-mortem studies have shown strong correlations between regional demyelinating lesion load and axonal density in the corresponding projection region of the CC in MS patients [40]. In agreement with that, we found a positive correlation between atrophy of the CC and whole brain lesion load. However, the correlation between iSP and atrophy but not with lesion load would suggest that the iSP onset latency reflects abnormalities beyond the visible white matter lesions. In addition, the results of our study add a piece of evidence on the close link between microstructure and function, showing a significant but moderate correlation between the iSP and MD and RD metrics. Nevertheless, the iSP abnormality seems to be able to capture signs of cognitive and physical impairment in MS that could be related to callosal disconnection.

A limitation of our study is the low number of patients included that might have potentially affected the statistical power. However, we studied a relatively homogeneous cohort of relapsing-remitting patients with non-disabling MS as their median EDSS score was 2.0 after mean disease duration longer than 5 years [33]. Moreover, the exploratory nature of this study precluded multiple testing correction analysis. However, the biological plausibility and the consistency of the results in agreement with previous research give further support to the conclusions. Nevertheless, it would be interesting to confirm the results of this study in a larger cohort including multiple testing correction analysis. Finally, we did not perform DTI tractography analysis to further characterize the relationship between the iSP and the topographical distribution of the CC abnormalities. Although this approach would be interesting to better understand the involved pathophysiology, it is likely that the regional distribution would not reach stronger correlations considering the large variability of both the extent and location of MS lesions.

In conclusion, we found evidence for structural and microstructural abnormalities of the CC that are associated with impairment of motor callosal inhibitory conduction in MS. CC damage is likely to contribute to cognitive dysfunction and in less extent to physical disability through a disconnection mechanism.
